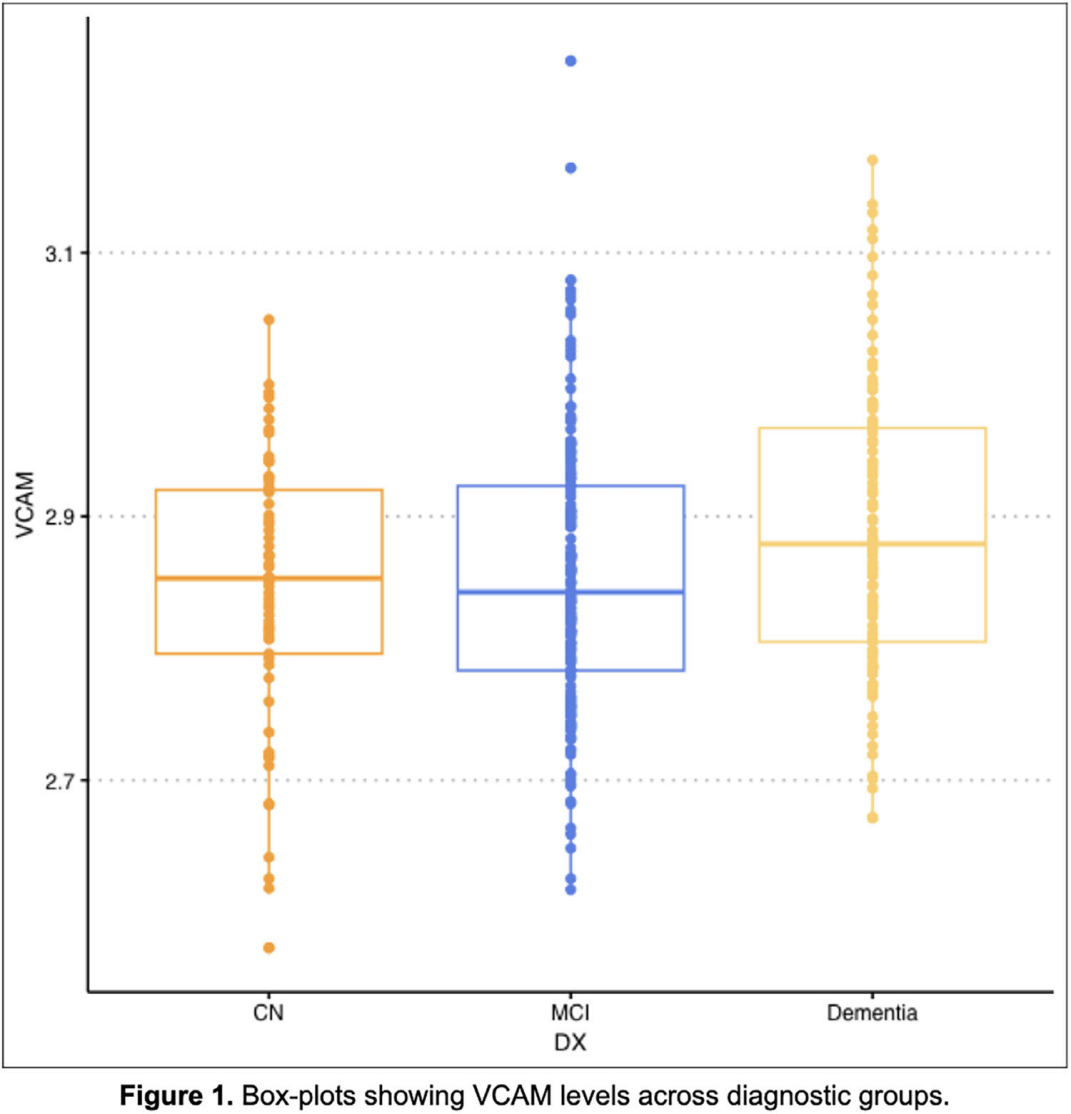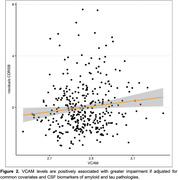# VCAM is associated with impairment in individuals with amyloid and tau pathology

**DOI:** 10.1002/alz.093447

**Published:** 2025-01-09

**Authors:** Pedro Rodrigues Vidor, Thomas Hugentobler Schlickmann, Luiza Santos Machado, Andreia Silva da Rocha, João Pedro Ferrari‐Souza, Eduardo R. Zimmer

**Affiliations:** ^1^ Universidade Federal do Rio Grande do Sul, Porto Alegre, Rio Grande do Sul Brazil; ^2^ University of Pittsburgh, Pittsburgh, PA USA; ^3^ Universidade Federal do Rio Grande do Sul, Porto Alegre Brazil; ^4^ McGill University, Montreal, QC Canada; ^5^ Brain Institute of Rio Grande do Sul ‐ Pontifícia Universidade Católica do Rio Grande do Sul, Porto Alegre, Rio Grande do Sul Brazil

## Abstract

**Background:**

Alzheimer's disease (AD) has been known for more than a century, but its complex pathophysiology remains unclear. Previous studies have been suggesting a potential role of neuroinflammation and cerebral vascular changes in AD progression. Part of the immune response relies on the role of Vascular Cell Adhesion Molecule (VCAM) in cell transit through the endothelium. However, there is little information about the impact of VCAM in AD‐related impairment. Here, we investigated the association of plasma VCAM levels with biological and clinical AD markers.

**Method:**

We assessed 357 individuals from the Alzheimer's Disease Neuroimaging Initiative cohort with plasma VCAM and medical data available. Statistical analyses were performed using R Studio. Association with diagnosis was evaluated by a linear regression between VCAM and clinical diagnosis, with adjustments for age, sex, *APOEε4* status and years of education. Regressions analysis was also used to assess the association of VCAM with Clinical Dementia Rating Scale ‐ Sum of Boxes (CDR‐SB), adjusting for age, sex, *APOEε4* status and years of education, as well as cerebrospinal fluid Aβ42 and p‐Tau181.

**Result:**

The group of individuals with dementia had higher blood VCAM levels (p=0.005) than both CN and MCI (p=0.003) groups, but there was no statistical significance between CN and MCI (Figure 1). VCAM showed a positive association (p = 0.018, Figure 2) with greater impairment as measured by CDR‐SB when adjustments for Aβ42 and p‐Tau181 were included.

**Conclusion:**

Our results suggest differential effects of vascular factors in biological and clinical AD. Further studies are needed to assess whether these relations include causality and whether targeting VCAM can lead to improvements in neuroinflammatory or vascular‐related changes.